# 2,6-Bis(tosyl­oxymeth­yl)pyridine

**DOI:** 10.1107/S160053681100050X

**Published:** 2011-01-08

**Authors:** Lynette Komarsamy, Muhammad D. Bala, Holger B. Friedrich, Bernard Omondi

**Affiliations:** a School of Chemistry, University of KwaZulu-Natal, Westville Campus, Private Bag X54001, Durban 4000, South Africa; b Research Centre for Synthesis and Catalysis, Department of Chemistry, University of Johannesburg, PO Box 524, Auckland Park, Johannesburg 2006, South Africa

## Abstract

The title compound, C_21_H_21_NO_6_S_2_, is organized around a twofold axis parallel to the crystallographic *c* axis and containing the N atom and a C atom of the pyridine ring. The tosyl moiety and the pyridine ring are both essentially planar [maximum deviations 0.028 (2) and 0.020 (3) Å, respectively]; their mean planes form a dihedral angle of 33.0 (2)°.

## Related literature

For related structures, see: Sellmann *et al.* (1999 ▶); Teixidor *et al.* (1999 ▶, 2001 ▶); Smit *et al.* (2004 ▶); Gilbert *et al.* (2000 ▶). For the synthesis of the title compound, see: Reger *et al.* (2005 ▶).
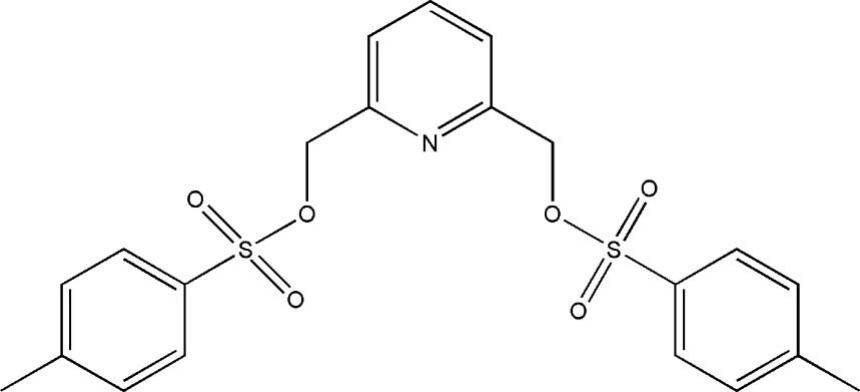


## Experimental

### 

#### Crystal data

C_21_H_21_NO_6_S_2_
*M* *_r_* = 447.51
Orthorhombic, 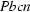

*a* = 21.032 (3) Å
*b* = 6.2243 (10) Å
*c* = 15.405 (2) Å
*V* = 2016.6 (5) Å^3^
*Z* = 4
Mo *K*α radiation
μ = 0.30 mm^−1^
*T* = 100 K
0.16 × 0.13 × 0.04 mm

#### Data collection

Bruker X8 APEXII 4K KappaCCD diffractometer
Absorption correction: multi-scan (*SADABS*; Bruker, 2009 ▶) *T*_min_ = 0.953, *T*_max_ = 0.988
41584 measured reflections
2528 independent reflections
1905 reflections with *I* > 2σ(*I*)
*R*_int_ = 0.097

#### Refinement

*R*[*F* ^2^ > 2σ(*F* ^2^)] = 0.047
*wR*(*F* ^2^) = 0.137
*S* = 0.96
2528 reflections
138 parameters
H-atom parameters constrained
Δρ_max_ = 0.63 e Å^−3^
Δρ_min_ = −0.43 e Å^−3^

### 

Data collection: *APEX2* (Bruker, 2009 ▶); cell refinement: *SAINT-Plus* (Bruker, 2009 ▶); data reduction: *SAINT-Plus* and *XPREP* (Bruker, 2009 ▶); program(s) used to solve structure: *SHELXS97* (Sheldrick, 2008 ▶); program(s) used to refine structure: *SHELXL97* (Sheldrick, 2008 ▶); molecular graphics: *ORTEP-3* (Farrugia, 1997 ▶); software used to prepare material for publication: *WinGX* (Farrugia, 1999 ▶).

## Supplementary Material

Crystal structure: contains datablocks global, I. DOI: 10.1107/S160053681100050X/dn2643sup1.cif

Structure factors: contains datablocks I. DOI: 10.1107/S160053681100050X/dn2643Isup2.hkl

Additional supplementary materials:  crystallographic information; 3D view; checkCIF report

## Figures and Tables

**Fig. 1.**
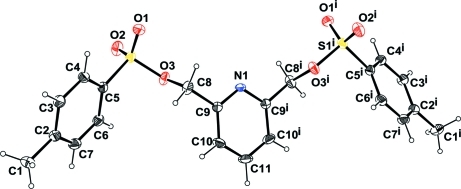
Molecular structure of the title compound with the atom numbering scheme. Displacement ellipsoids are drawn at the 50% probability level. H atoms are presented as small spheres of arbitrary radius. [Symmetry code: (i) -x, y, -z + 3/2].

## supplementary crystallographic information

### Comment

The title compound, (I), is commonly used as a very convenient precursor for the synthesis of a variety of pyridine containing compounds some of which have been highlighted by Reger *et al.*, 2005. Our investigation of the use of this compound as a precursor to the synthesis of tridentate pyridine containing SNS ligands has lead to the determination of its crystal structure contained in this report.

The compound is organized around a two fold axis containing the N1 and C11 atoms. The two tosyl groups are nearly orthogonal about the pyridyl moiety with an N1—C9—C8—O3 torsion angle of 77.3° and in addition the tosyl moiety was found to be planar with C5 and S1 deviating the most from the plane by 0.023 (3) Å and 0.028 (2) Å respectively. The five atoms of the pyridine ring lie on a plane with atom C10 showing the most deviation of 0.020 (3) Å from this plane. The axes of the planes of the two moieties (tosyl and pyridyl) intersect at a very acute angle of 33.0°.

### Experimental

The title compound, 2,6-bis(tosylmethyl)pyridne (I) was synthesized by the adaptation of a modified literature method (Reger *et al.*, 2005). To a 500 ml round bottom flask NaOH (8.0 g, 0.20 mol) and pyridine dimethanol (2.78 g, 0.20 mol) was dissolved in 150 ml THF/water (1:1). To this stirred solution a solution of *p*-toluenesulfonyl chloride in THF (75 ml) (7.61 g, 0.040 mol) was added at 0 °C and the reaction mixture was left to stir for about 15 min at 0 °C and then at room temperature for a total time of 4 h. The mixture was then poured into 200 ml of water and extracted with dichloromethane (4 *x* 75 ml). The organic phase was washed with a saturated solution of NaCl and dried using Na_2_SO_4_ and the solvent was removed *in vacuo* to produce the resulting product as a white crystalline solid (7.12 g, 80%). Single crystals were obtained by dissolving the product, (I), in THF and ethanol and allowing the solvents to evaporate slowly at room temperature in air. Spectroscopic data: ^1^H NMR (400 MHz, CDCl_3_, δ. p.p.m.): = 2.4 (s, 6H), 5.1 (s, 4H), 7.3 (d, 6H), 7.7 (t, 1H), 7.8 (d, 4H). FT—IR (cm^-1^): 3068(w), (C=C), 2958(w), (CH_3_,CH2), 1596(*m*), (ar), 1167(*s*), (C—O), 1028(*m*), (S=O).

### Refinement

All H-atoms were refined using a riding model, with C—H = 0.93 Å and *U*_iso_(H) = 1.2*U*_eq_(C) for aromatic, C—H = 0.97 Å and *U*_iso_(H) = 1.2*U*_eq_(C) for CH_2_, C—H = 0.96 Å and *U*_iso_(H) = 1.5*U*_eq_(C) for CH_3_.

### Crystal data

**Table:**

C_21_H_21_NO_6_S_2_	*F*(000) = 936
*M**_r_* = 447.51	*D*_x_ = 1.474 Mg m^−^^3^
Orthorhombic, *P**b**c**n*	Mo *K*α radiation, λ = 0.71073 Å
Hall symbol: -P 2n 2ab	Cell parameters from 45194 reflections
*a* = 21.032 (3) Å	θ = 1.9–28.4°
*b* = 6.2243 (10) Å	µ = 0.30 mm^−^^1^
*c* = 15.405 (2) Å	*T* = 100 K
*V* = 2016.6 (5) Å^3^	Plate, colourless
*Z* = 4	0.16 × 0.13 × 0.04 mm

### Data collection

**Table:**

Bruker X8 APEXII 4K KappaCCD diffractometer	2528 independent reflections
Radiation source: fine-focus sealed tube	1905 reflections with *I* > 2σ(*I*)
graphite	*R*_int_ = 0.097
φ and ω scans	θ_max_ = 28.4°, θ_min_ = 1.9°
Absorption correction: multi-scan (*SADABS*; Bruker, 2009)	*h* = −28→28
*T*_min_ = 0.953, *T*_max_ = 0.988	*k* = −8→8
41584 measured reflections	*l* = −20→20

### Refinement

**Table:**

Refinement on *F*^2^	Primary atom site location: structure-invariant direct methods
Least-squares matrix: full	Secondary atom site location: difference Fourier map
*R*[*F*^2^ > 2σ(*F*^2^)] = 0.047	Hydrogen site location: inferred from neighbouring sites
*wR*(*F*^2^) = 0.137	H-atom parameters constrained
*S* = 0.96	*w* = 1/[σ^2^(*F*_o_^2^) + (0.0723*P*)^2^ + 3.1627*P*] where *P* = (*F*_o_^2^ + 2*F*_c_^2^)/3
2528 reflections	(Δ/σ)_max_ = 0.001
138 parameters	Δρ_max_ = 0.63 e Å^−^^3^
0 restraints	Δρ_min_ = −0.43 e Å^−^^3^

### Special details

**Table:**

Experimental. The intensity data was collected on a Bruker X8 Apex 4 K CCD diffractometer using an exposure time of 20 sec/per frame. A total of 2647 frames were collected with a frame width of 0.5° covering upto θ = 28.38° with 99.8% completeness accomplished.
Geometry. All e.s.d.'s (except the e.s.d. in the dihedral angle between two l.s. planes) are estimated using the full covariance matrix. The cell e.s.d.'s are taken into account individually in the estimation of e.s.d.'s in distances, angles and torsion angles; correlations between e.s.d.'s in cell parameters are only used when they are defined by crystal symmetry. An approximate (isotropic) treatment of cell e.s.d.'s is used for estimating e.s.d.'s involving l.s. planes.
Refinement. Refinement of *F*^2^ against ALL reflections. The weighted *R*-factor *wR* and goodness of fit *S* are based on *F*^2^, conventional *R*-factors *R* are based on *F*, with *F* set to zero for negative *F*^2^. The threshold expression of *F*^2^ > σ(*F*^2^) is used only for calculating *R*-factors(gt) *etc*. and is not relevant to the choice of reflections for refinement. *R*-factors based on *F*^2^ are statistically about twice as large as those based on *F*, and *R*- factors based on ALL data will be even larger.

### Fractional atomic coordinates and isotropic or equivalent isotropic displacement parameters (Å^2^)

**Table:**

	*x*	*y*	*z*	*U*_iso_*/*U*_eq_	
C1	0.18962 (13)	0.4605 (4)	0.24649 (17)	0.0264 (5)	
H1A	0.2242	0.5224	0.2120	0.040*	
H1B	0.1517	0.4455	0.2101	0.040*	
H1C	0.2024	0.3190	0.2682	0.040*	
C2	0.17507 (11)	0.6057 (4)	0.32188 (16)	0.0197 (5)	
C3	0.14727 (11)	0.8063 (4)	0.30711 (15)	0.0202 (5)	
H3	0.1383	0.8505	0.2494	0.024*	
C4	0.13258 (11)	0.9415 (4)	0.37560 (15)	0.0187 (5)	
H4	0.1129	1.0764	0.3652	0.022*	
C5	0.14700 (11)	0.8778 (4)	0.45964 (15)	0.0174 (4)	
C6	0.17551 (11)	0.6802 (4)	0.47619 (16)	0.0198 (5)	
H6	0.1854	0.6378	0.5339	0.024*	
C7	0.18916 (11)	0.5467 (4)	0.40679 (16)	0.0212 (5)	
H7	0.2086	0.4115	0.4174	0.025*	
C8	0.03481 (11)	0.8393 (4)	0.60090 (16)	0.0217 (5)	
H8A	0.0330	0.7504	0.5477	0.026*	
H8B	0.0061	0.9640	0.5936	0.026*	
C9	0.01580 (10)	0.7094 (4)	0.67889 (15)	0.0169 (5)	
C10	0.01592 (12)	0.4865 (4)	0.67603 (18)	0.0238 (5)	
H10	0.0268	0.4128	0.6241	0.029*	
C11	0.0000	0.3742 (6)	0.7500	0.0283 (8)	
H11	0.0000	0.2216	0.7500	0.034*	
N1	0.0000	0.8211 (4)	0.7500	0.0162 (5)	
O1	0.08679 (8)	1.2125 (3)	0.51596 (12)	0.0240 (4)	
O2	0.18953 (9)	1.1249 (3)	0.58438 (12)	0.0272 (4)	
O3	0.09992 (8)	0.9111 (3)	0.61752 (11)	0.0207 (4)	
S1	0.13181 (3)	1.05596 (9)	0.54528 (4)	0.01895 (17)	

### Atomic displacement parameters (Å^2^)

**Table:**

	*U*^11^	*U*^22^	*U*^33^	*U*^12^	*U*^13^	*U*^23^
C1	0.0318 (13)	0.0197 (12)	0.0277 (14)	0.0030 (10)	−0.0001 (11)	−0.0062 (10)
C2	0.0197 (11)	0.0173 (11)	0.0220 (12)	−0.0019 (8)	0.0010 (9)	−0.0011 (9)
C3	0.0255 (11)	0.0210 (11)	0.0142 (11)	0.0015 (9)	−0.0002 (9)	0.0022 (9)
C4	0.0228 (11)	0.0167 (10)	0.0166 (11)	0.0027 (9)	0.0012 (9)	0.0022 (9)
C5	0.0190 (10)	0.0173 (10)	0.0158 (11)	−0.0014 (8)	0.0029 (8)	−0.0010 (8)
C6	0.0222 (11)	0.0195 (11)	0.0176 (11)	−0.0007 (9)	0.0006 (9)	0.0049 (9)
C7	0.0213 (11)	0.0166 (11)	0.0256 (13)	0.0014 (9)	−0.0009 (9)	0.0028 (9)
C8	0.0205 (11)	0.0274 (12)	0.0172 (12)	−0.0062 (9)	0.0008 (9)	−0.0005 (9)
C9	0.0174 (10)	0.0156 (10)	0.0176 (11)	−0.0009 (8)	0.0018 (8)	−0.0011 (8)
C10	0.0249 (12)	0.0155 (11)	0.0310 (14)	0.0006 (9)	0.0021 (10)	−0.0084 (9)
C11	0.0277 (18)	0.0107 (14)	0.046 (2)	0.000	0.0005 (16)	0.000
N1	0.0215 (13)	0.0095 (12)	0.0175 (13)	0.000	0.0019 (11)	0.000
O1	0.0306 (9)	0.0174 (8)	0.0242 (9)	0.0013 (7)	0.0062 (7)	−0.0012 (7)
O2	0.0273 (9)	0.0335 (10)	0.0208 (9)	−0.0112 (8)	0.0030 (7)	−0.0054 (8)
O3	0.0213 (8)	0.0260 (9)	0.0147 (8)	−0.0065 (7)	0.0019 (6)	0.0018 (7)
S1	0.0225 (3)	0.0192 (3)	0.0152 (3)	−0.0044 (2)	0.0036 (2)	−0.0017 (2)

### Geometric parameters (Å, °)

**Table:**

C1—C2	1.503 (3)	C8—O3	1.463 (3)
C1—H1A	0.9800	C8—C9	1.502 (3)
C1—H1B	0.9800	C8—H8A	0.9900
C1—H1C	0.9800	C8—H8B	0.9900
C2—C7	1.391 (3)	C9—N1	1.339 (3)
C2—C3	1.397 (3)	C9—C10	1.388 (3)
C3—C4	1.385 (3)	C10—C11	1.378 (3)
C3—H3	0.9500	C10—H10	0.9500
C4—C5	1.388 (3)	C11—C10^i^	1.378 (3)
C4—H4	0.9500	C11—H11	0.9500
C5—C6	1.392 (3)	N1—C9^i^	1.339 (3)
C5—S1	1.753 (2)	O1—S1	1.4315 (18)
C6—C7	1.384 (3)	O2—S1	1.4217 (19)
C6—H6	0.9500	O3—S1	1.5816 (17)
C7—H7	0.9500		
			
C2—C1—H1A	109.5	O3—C8—C9	105.87 (18)
C2—C1—H1B	109.5	O3—C8—H8A	110.6
H1A—C1—H1B	109.5	C9—C8—H8A	110.6
C2—C1—H1C	109.5	O3—C8—H8B	110.6
H1A—C1—H1C	109.5	C9—C8—H8B	110.6
H1B—C1—H1C	109.5	H8A—C8—H8B	108.7
C7—C2—C3	118.6 (2)	N1—C9—C10	123.0 (2)
C7—C2—C1	121.6 (2)	N1—C9—C8	116.1 (2)
C3—C2—C1	119.8 (2)	C10—C9—C8	120.8 (2)
C4—C3—C2	120.8 (2)	C11—C10—C9	118.7 (2)
C4—C3—H3	119.6	C11—C10—H10	120.6
C2—C3—H3	119.6	C9—C10—H10	120.6
C3—C4—C5	119.2 (2)	C10—C11—C10^i^	119.0 (3)
C3—C4—H4	120.4	C10—C11—H11	120.5
C5—C4—H4	120.4	C10^i^—C11—H11	120.5
C4—C5—C6	121.2 (2)	C9—N1—C9^i^	117.4 (3)
C4—C5—S1	118.78 (18)	C8—O3—S1	116.61 (15)
C6—C5—S1	119.99 (18)	O2—S1—O1	119.53 (11)
C7—C6—C5	118.6 (2)	O2—S1—O3	103.66 (10)
C7—C6—H6	120.7	O1—S1—O3	109.24 (10)
C5—C6—H6	120.7	O2—S1—C5	110.76 (11)
C6—C7—C2	121.6 (2)	O1—S1—C5	108.28 (11)
C6—C7—H7	119.2	O3—S1—C5	104.25 (10)
C2—C7—H7	119.2		
			
C7—C2—C3—C4	−1.6 (4)	C9—C10—C11—C10^i^	0.47 (16)
C1—C2—C3—C4	179.0 (2)	C10—C9—N1—C9^i^	0.52 (17)
C2—C3—C4—C5	1.3 (4)	C8—C9—N1—C9^i^	−178.6 (2)
C3—C4—C5—C6	−0.4 (4)	C9—C8—O3—S1	−179.17 (15)
C3—C4—C5—S1	176.85 (18)	C8—O3—S1—O2	171.80 (17)
C4—C5—C6—C7	−0.2 (3)	C8—O3—S1—O1	43.33 (19)
S1—C5—C6—C7	−177.48 (18)	C8—O3—S1—C5	−72.23 (18)
C5—C6—C7—C2	0.0 (4)	C4—C5—S1—O2	−113.4 (2)
C3—C2—C7—C6	0.9 (4)	C6—C5—S1—O2	64.0 (2)
C1—C2—C7—C6	−179.7 (2)	C4—C5—S1—O1	19.5 (2)
O3—C8—C9—N1	77.3 (2)	C6—C5—S1—O1	−163.18 (18)
O3—C8—C9—C10	−101.8 (3)	C4—C5—S1—O3	135.74 (19)
N1—C9—C10—C11	−1.0 (3)	C6—C5—S1—O3	−47.0 (2)
C8—C9—C10—C11	178.03 (18)		

Symmetry codes: (i) −*x*, *y*, −*z*+3/2.
